# Transient Elastography and Video Recovery Narrative Access to Support Recovery From Alcohol Misuse: Development of a Novel Intervention for Use in Community Alcohol Treatment Services

**DOI:** 10.2196/47109

**Published:** 2023-10-04

**Authors:** Stefan Rennick-Egglestone, Mohsan Subhani, Holly Knight, Katy A Jones, Clare Hutton, Tracey Jackson, Matthew Hutton, Andrew Wragg, Joanne R Morling, Kirsty Sprange, Stephen D Ryder

**Affiliations:** 1 School of Health Sciences Institute of Mental Health University of Nottingham Nottingham United Kingdom; 2 Nottingham Digestive Diseases Biomedical Research Centre School of Medicine University of Nottingham Nottingham United Kingdom; 3 NIHR Nottingham Biomedical Research Centre Nottingham University Hospitals NHS Trust and the University of Nottingham Nottingham United Kingdom; 4 Lifespan and Population Health School of Medicine University of Nottingham Nottingham United Kingdom; 5 Academic Unit of Mental Health and Clinical Neuroscience School of Medicine, Institute of Mental Health University of Nottingham Nottingham United Kingdom; 6 KLIFAD Study PPI Panel Nottingham United Kingdom; 7 Nottingham Clinical Trials Research Unit University of Nottingham Nottingham United Kingdom

**Keywords:** recovery narrative, recovery story, alcohol misuse, alcohol use disorder, feasibility trial, complex intervention, KLIFAD intervention

## Abstract

**Background:**

Mortality from alcohol-related liver disease has risen significantly for 3 decades. Transient elastography (TE) is a noninvasive test providing a numerical marker of liver disease. Preliminary evidence suggests that TE can reduce alcohol consumption. The KLIFAD (does knowledge of liver fibrosis affect high-risk drinking behavior?) study has developed a complex intervention wherein people receiving alcohol treatment are provided with access to TE, accompanied by scripted feedback tailored to their disease state, and access to video narratives describing alcohol misuse recovery after receiving TE. Recovery narratives are included due to preliminary evidence from mental health studies which suggest that access to digital narratives describing recovery from mental health problems can help people affected by mental health problems, including through mechanisms with the potential to be transferable to an alcohol treatment setting, for example, by increasing hope for the future, enabling learning from the experience of others, or promoting help-seeking behaviors.

**Objective:**

We aimed to develop the KLIFAD intervention to the point that it could be delivered in a feasibility trial and to produce knowledge relevant to clinicians and researchers developing interventions making use of biomarkers of disease.

**Methods:**

In research activity 1, standardized scripted feedback was developed by this study, and then iterated through focus groups with people who had experienced alcohol misuse and TE, and key alcohol workers with experience in delivering TE. We report critical design considerations identified through focus groups, in the form of sensitizing concepts. In research activity 2, a video production guide was coproduced to help produce impactful video-based recovery narratives, and a patient and public involvement (PPI) panel was consulted for recommendations on how best to integrate recovery narratives into an alcohol treatment setting. We report PPI recommendations and an overview of video form and content.

**Results:**

Through research activity 1, we learnt that patient feedback has not been standardized in prior use of TE, that receiving a numeric marker can provide an objective target that motivates and rewards recovery, and that key alcohol workers regularly tailor information to their clients. Through research activity 2, we developed a video production guide asking narrators what recovery means to them, what helped their recovery, and what they have learned about recovery. We produced 10 recovery narratives and collected PPI recommendations on maximizing impact and safety. These led to the production of unplanned videos presenting caregiver and clinician perspectives, and a choice to limit narrative availability to alcohol treatment settings, where support is available around distressing content. These choices have been evaluated through a feasibility randomized controlled trial [ISRCTN16922410].

**Conclusions:**

Providing an objective target that motivates and rewards recovery is a candidate change mechanism for complex interventions integrating biomarkers of disease. Recovery narratives can contain distressing content; intervention developers should attend to safe usage.

**International Registered Report Identifier (IRRID):**

RR2-10.1136/bmjopen-2021-054954

## Introduction

### Background

Mortality from alcohol-related liver disease (ARLD) has risen significantly over the past 3 decades, and it is now the second most common cause of working life years lost in men and the fifth in women [1]. While ARLD is caused by alcohol misuse, there is currently no consistent definition of this term, and henceforth, we consider that alcohol misuse is indicated by any of the following [2]: a weekly alcohol intake of 14 units or greater; a score of 8 or greater on the Alcohol Use Disorder Identification Test [3]; or a referral by a health care worker to a specialist alcohol treatment service.

Harmful consumption of alcohol increases fat accumulation in the liver. This triggers an inflammatory response, which eventually causes scarring of the liver, known as liver fibrosis. Without intervention, liver fibrosis can progress to cirrhosis and can ultimately result in liver failure. ARLD develops silently, often exhibiting no symptoms until complications occur. A large population study using UK clinical records found that 47.3% of cases (2420 of 5118) were first detected following an emergency presentation to a hospital with end-stage (decompensated) liver disease [4].

Early diagnosis of liver fibrosis provides the best opportunity to impact harmful drinking behaviors and hence prevent cirrhosis. Traditionally, liver biopsies have been seen as the “gold standard” for diagnosis, but the potential of liver biopsy to facilitate early diagnosis at scale is limited by its invasiveness and associated resource implications [5]. Noninvasive diagnostic technologies are now available, including transient elastography (TE), in which an ultrasound probe is placed on the surface of the skin and used to measure liver stiffness as a proxy for liver health [5]. Early studies indicated that TE is not operator-dependent, has good overall accuracy in diagnosing advanced fibrosis and cirrhosis regardless of underlying etiology, and has moderate accuracy in diagnosing milder fibrosis [5]. For 1 study population, using TE to screen asymptomatic individuals in a community setting doubled the rate of liver cirrhosis diagnosis [6]. In the United Kingdom, the National Institute for Health and Care Excellence recommends the use of TE to diagnose cirrhosis in individuals with harmful alcohol use or diagnosed with ARLD, with supervision from a competent user for around the first 50 uses [7].

As well as enabling referral into treatment pathways, receiving TE has the potential to reduce alcohol use directly, and hence to be used as an active ingredient in novel interventions for alcohol misuse. A nonrandomized study in a UK community setting presented preliminary evidence that receiving a TE through FibroScan (a specific form of TE) reduced alcohol use without referral to treatment [8]. FibroScan delivers a single score quantifying liver stiffness and therefore damage. In this study, participants with scores indicating raised liver stiffness experienced greater reductions in subsequent alcohol use than those with scores indicating normal liver stiffness. There was no evidence of false reassurance in the latter [8]. A systematic review and meta-analysis of studies providing advice to patients based on numeric markers of liver injury demonstrated greater alcohol reduction in the intervention compared to control (nonspecific advice) conditions. This study highlighted a lack of standardization in advice content and delivery [9]. TE can be delivered briefly, as the scanning process is rapid, and results are available immediately after the scan. A systematic review has demonstrated that brief alcohol interventions reduce alcohol consumption in harmful drinkers [10].

### Development of the “does knowledge of liver fibrosis affect high-risk drinking behavior?” Intervention

This paper reports on the development of the KLIFAD (does knowledge of liver fibrosis affect high-risk drinking behavior?) intervention, a complex intervention with a novel digital component, which is intended for use in UK community alcohol services. The KLIFAD intervention was developed by the KLIFAD study (National Institute for Health and Care Research, Research for Patient Benefit programme, NIHR201146).

In the KLIFAD intervention, service users receive TE by FibroScan in an established alcohol treatment setting, accompanied by (1) brief advice about the meaning of the numerical scan result and its relationship to their liver health and alcohol use and (2) access to a collection of prerecorded video narratives in which real narrators (ie, people who are describing their own experiences and who are not actors) describe their recovery from alcohol misuse after receiving TE (henceforth recovery narratives). Brief advice is delivered in the form of standardized scripted feedback, first explained to the patient by a formally trained FibroScan operator, and then provided in the form of a printed sheet. The brief advice used in the KLIFAD intervention was developed with people with lived experience of excess alcohol use and of receiving a FibroScan. The FibroScan operator is a trained health care professional, and can also refer recipients to other workers in the setting if further support is required, for example, if strong emotions are experienced when receiving results indicating liver disease.

Video-based recovery narratives were included in the KLIFAD intervention due to preliminary evidence from mental health studies that they can create helpful change in recipients experiencing mental health problems. This change takes place through mechanisms that have the potential to be transferable to an alcohol treatment setting, for example, by increasing hope for the future, enabling learning from the experience of others, decreasing internalized stigma (self-stigma), or promoting help-seeking behaviors [11-13]. Together, operator training material, scripted feedback, and the recovery narrative collection form a tightly specified complex intervention. Our usage of recovery narratives is in keeping with a growing body of work in which recovery narratives are treated as active ingredients in digital health interventions across a range of health conditions.

In keeping with Medical Research Council guidance on developing and evaluating complex interventions [14], KLIFAD has evaluated (1) the feasibility of integrating the KLIFAD intervention into community alcohol treatment services in England and (2) the feasibility of conducting a definitive randomized controlled trial (RCT) of the KLIFAD intervention in this setting. The evaluation was through a 2-arm feasibility RCT (ISRCTN16922410) [2]. Screened participants who had recently been allocated to a defined alcohol treatment program delivered by the treatment service were offered trial participation and continued with their allocated treatment. Consented participants were randomized (intervention; control). Intervention arm participants additionally received the KLIFAD intervention early in their treatment. Findings from this trial indicated a definitive national RCT should be conducted in the same setting. Compared to control, intervention arm participants had an increased likelihood of treatment program completion and a reduction in self-reported alcohol consumption [15].

The aim of this paper is to describe 2 formative research studies that were conducted to define the KLIFAD intervention for the purposes of the KLIFAD trial. A process evaluation for the KLIFAD trial will be reported elsewhere and will describe participant experiences of the KLIFAD intervention as deployed in the KLIFAD trial. Our formative studies had a meaningful impact on how the KLIFAD intervention was deployed and delivered. Our intention in describing the development process and our learning from it is to provide transparency for critical decisions that were made for the KLIFAD trial. Our findings will also support the modification of the KLIFAD intervention to settings other than English community alcohol services. We also want to share knowledge to support the development of related complex interventions more broadly, such as those that incorporate the use of biomarkers of disease to enable health-related behavior change outside of alcohol misuse.

## Methods

### Patient and Public Involvement

The research described in this paper was guided by a patient and public involvement (PPI) panel who met regularly during the work. The PPI panel had 5 members, all with lived experience of alcohol misuse and treatment. Some panel members had lived experience of receiving a FibroScan during treatment and could describe a substantial personal impact on their recovery of receiving FibroScan scores. Some had lived experience of caring for others misusing alcohol. The work of the panel was facilitated by a chair (AW). Panel members made a substantial contribution to the envisioning and success of intervention development work and were offered the opportunity to meet the International Committee of Medical Journal Editors criteria on authorship for this paper. Further, 3 (CH, TJ, and MH) opted to meet the International Committee of Medical Journal Editors criteria and are included in the authorship list. Some other authors have experience of alcohol misuse and of receiving treatment.

### Study Summary

With our PPI panel, we identified 2 critical components of the intervention to focus on through numbered research activities (RAs) with defined research methods: (1) RA1: design of standardized scripted feedback and (2) RA2: design of video recovery narratives

Each RA had a dual purpose: (1) to develop aspects of the KLIFAD intervention to the point that it could be delivered in a feasibility trial and (2) to produce knowledge relevant to clinicians and researchers engaged in intervention development work.

RA1 was operationalized through focus groups that iteratively developed preliminary prototypes of scripted feedback. For RA2, we created a video production guide in collaboration with the PPI panel and worked with a videographer to produce videos for inclusion in the KLIFAD trial. Methodological details are provided later in this section.

### Ethical Considerations

Ethical approval for conducting the 2 RAs was obtained in advance from a UK National Health Service Research Ethics Committee (West of Scotland Research Ethics Service, REC reference: 20/WS/0179).

Focus group participants were provided with a participant information sheet at the first focus group they attended. They provided written informed consent before the focus group started. Consent was reconfirmed verbally at subsequent focus groups for those participants who attended multiple focus groups. Participation in focus groups was confidential. Transcripts were pseudonymized before analysis. Only the principal investigator and delegates retained access to verbatim transcripts, which were deleted before this study’s end.

Video narrators were treated as research participants, and gave written informed consent before the video production process began. The informed consent process included the formal provision of a participant information sheet and as much discussion as was needed with this study’s coordinator (MS) and the videographer to ensure that participants understood how their video would be used and what it might feel like to appear in a video recovery narrative.

As well as being a form of research data, videos were intended for use in the KLIFAD intervention and hence were watched by some participants in the KLIFAD trial. Narrators of videos were given the choice to reveal or to occlude their identity in their video. If identity occlusion was desired, the method was agreed with the videographer, who documented and enacted it. Examples included blurring the face of a narrator. Our decision to give narrators control over the presentation of their identity was in keeping with an ethical principle developed by a prior study that has developed a mental health recovery narrative collection [16]. This principle was informed by a systematic review and interview study on considerations in recovery narrative collection practices [17,18].

For both RAs, consent records have been retained by this study’s sponsor, for the retention period specified by this study’s sponsor.

### RA1: Development of Standardized Scripted Feedback

#### Overview

In RA1, we worked with people with lived experience of receiving TE to develop an initial version of the standardized scripted feedback used in the KLIFAD intervention. These are referred to as experts by experience (EBE). We then validated this scripted feedback through consultation with trained FibroScan operators employed as key alcohol workers (KAW).

#### Participants

Inclusion criteria for EBE: 18 years or older, current or prior experience of alcohol misuse, and had previously undergone TE by FibroScan. Exclusion criteria: other substance misuse was present. Inclusion criteria for KAW: 18 years or older, employed at the 2 planned principal trial recruitment sites, and have previously delivered FibroScans.

The principal trial recruitment sites employing the KAW were (1) a city center drug and alcohol treatment service, which also provides support for mental health, housing, and employment, and (2) a 62-bed facility for adults who experience physical and mental health problems due to drug and Alcohol Use Disorder, providing a structured alcohol detox program along with mental health and social support.

#### Procedures

EBE were identified through (1) participant information sheets distributed to the leads of existing patient groups, (2) recruitment posters displayed in affiliated alcohol treatment settings, (3) a research recruitment web page, (4) emails sent to prior research participants who had provided appropriate consent-to-contact, and (5) through research participants recommending the study to others. Inclusion criteria were self-rated by EBE; clinical records were not examined to verify inclusion. Our target was to recruit 6-10 EBE. This target was based on prior team experience of facilitating focus groups and sought to strike a balance between the inclusion of a range of perspectives and the need to provide individuals with temporal space to meaningfully contribute. No demographic information was collected for EBE.

KAW were recruited through site managers. Our target was to recruit 2 KAW from each trial recruitment site so as to access a mixture of perspectives from both sites. No demographic information was collected for KAW.

To prototype different ways of providing and displaying feedback on FibroScan results, we consulted general evidence-based recommendations regarding effective patient feedback [19], and the specific findings of a community-based liver disease detection study [20]. An initial template for scripted feedback was developed by a hepatology fellow (MS), a hepatology professor (SDR), and a qualitative researcher (HK). There are 3 broad categories of FibroScan score as defined by disease state: normal or no fibrosis (score <7); intermediate fibrosis (score 8-14); advanced fibrosis or cirrhosis (score >14). Hence, 3 pieces of scripted feedback were produced. Each sought to contextualize and explain the significance of liver damage as a prognostic indicator of future clinical outcomes. Each script was tailored to help avoid potential harms associated with the disease state, such as false reassurance for those who reported still drinking heavily, but whose score indicated no fibrosis or normal liver function.

We then hosted 3 focus groups to iteratively develop the prototype. Each was moderated by an experienced qualitative researcher (HK) with a second facilitator present (MS). Focus groups were audio-recorded and transcribed, initially by an automated transcription service, and then with subsequent validation and anonymization by a researcher. In focus group 1, 7 EBE were provided with a printed copy of the initial scripted feedback and were asked to provide feedback on readability, ease of comprehension, relevance of included information, and the best way to present the TE results. The interview guide is in Multimedia Appendix 1. Scripted feedback was redrafted based on group feedback. Focus group 2 was held 1 month later to review the iterated version. The same participants were invited. Further, 3 participants attended, and 2 sent email feedback. A proposed final version was produced and reviewed by 4 KAW in focus group 3 (interview guide in Multimedia Appendix 2).

#### Analysis

Pseudonymized focus group transcripts and contextual notes were analyzed using a pragmatic content analysis approach. To update scripted feedback, 2 researchers (MS and HK) triangulated participant feedback, and changes were made if they were supported by the majority of participants. MS and HK reflected on discrepant opinions to learn about the range of responses provided, to guide how findings are reported. We report critical design considerations identified through our analysis of focus group transcripts. In keeping with prior design-oriented research work [21], these are presented in the form of sensitizing concepts [22] selected for their broader relevance to the development of complex interventions featuring markers of illness.

### RA2: Production of Alcohol Misuse Recovery Narrative Videos

#### Overview

In RA2, we produced a collection of video-based recovery narratives for use in the KLIFAD intervention. This is referred to as the KLIFAD collection. Videos were intended to be impactful, for example, to have the capacity to create change in alcohol consumption behavior on the part of participants in the KLIFAD trial.

#### Participants

Recovery narrative videos featured participants (1) willing to be video-recorded, (2) aged 18 years or older, (3) with the capacity to consent, (4) who had previously received one or more TEs by FibroScan, (5) who could recall an approximate or exact value for their first score, (6) who attended with a primary problem of alcohol misuse as defined by initial clinical assessment, and (7) who was certain they had experienced recovery from their alcohol-use condition or had been identified as having experienced recovery by this study’s team.

#### Procedures

The recruitment approach was the same as for RA1. After informed consent was provided, demographic and clinical information was collected verbally on gender, age, ethnicity, estimated peak alcohol consumption, approximate first FibroScan score, and liver disease state at first diagnosis. Ethnicity was as identified by the participant (eg, there were no predefined categories). The number of recovery narrative videos (n=10) was predetermined by the scale of study resources for video production work. Recruitment work sought variations on gender, liver disease state at first diagnosis, and ethnicity. It prioritized variation in liver disease state.

Recovery narrative videos were produced by a professional videographer, in the form of short digital videos. To guide their work, a video production guide was coproduced by the PPI panel and research team and was provided to the videographer before their work began.

A prior study has indicated that mental health recovery narratives perceived as authentic are more likely to be impactful on recipients, and hence to determine preliminary content for the guide, a list of elements with the potential to enable a perception of authenticity was identified from study findings [12]. These were that the narrator talks about (1) their achievements in relation to alcohol use, (2) their difficulties in relation to alcohol use, (3) the impact of their alcohol use on others, (3) the strategies that they have successfully used to recover, (4) how thinking about alcohol use has changed, (5) the barriers to their recovery they have experienced, (6) the beliefs and values that have supported their recovery, and (7) their emotions around their alcohol use. Further, the narrator shares advice on how to use health services to support recovery.

These were operationalized into a 3-part video structure described in Textbox 1.

**Sample questions developed to inform video production work.**

**What does recovery mean to you?**

- What gives you hope?
- What makes you feel well?

**What has helped your recovery?**

- What was your first step on your recovery journey? What helped you take this step?
- What works for you and why?
- What activities have helped you? How do you feel when you are doing them? What has helped during times of hardship?
- At what point did you realise that you needed support?
- Where did you find the support? Was this challenging?
- Has there been someone who has supported you during your recovery journey? What were the barriers to recovery? How did you overcome them?
- What has been unhelpful or missing in your recovery?

**What have you learned about recovery?**

- Do you have any techniques that have been helpful when you are feeling really down?
- What sort of lessons would you like to pass onto others?
- If you could give one thing to assist someone’s recovery what would that be?
- What has helped you to build resilience?
- What would you tell someone who feels they won’t recover?
- How did you deal with changes to your recovery journey?

Guidance on how to create a positive and safe environment was integrated from existing guidance on the production of mental health recovery narratives [23]. A preliminary version of the guide was refined through a meeting of the PPI panel. The final version of the video production guide is included in Multimedia Appendix 3. The PPI panel made recommendations on the integration of videos into the KLIFAD intervention.

The videographer worked with participants to identify a setting for their video which was meaningful and respond to any personal concerns (eg, around disclosure of identity). They then recorded and produced preliminary versions of videos. These were inspected by a panel consisting of people with lived experience of alcohol misuse, a hepatology specialist, a PPI coordinator, and an academic expert in the use of recovery narratives to create change, who proposed refinements to the video. The opening screen described: age, estimated peak alcohol consumption, approximate first FibroScan score, and (where the narrator consented) included a photograph.

While all narrators had received a FibroScan, we chose not to require narrators to discuss their FibroScan and its impact. This was to allow narrators the freedom to discuss other issues if narrators considered these more consequential. In practice, all narrators discussed their FibroScan and its impact.

#### Analysis

Recommendations of the PPI meeting were captured in minutes and are reported in full. Videos were inspected, and an outline description of form and content was produced.

## Results

### RA1: Knowledge Produced Through Focus Groups

#### Overview

We present sensitizing concepts describing transferable knowledge developed through focus groups with EBE and KAW.

##### Sensitizing Concept 1: Historic Practices Around FibroScan Feedback Have not Been Standardized

Some EBE described being given their score directly after a scan:

I went into the room, I had my FibroScan. Straight away they said your number is 29 which is cirrhotic. You know you’ve got some form of liver disease. It was quite an eye opener for me.

Others were not given a score, but instead an ambiguous verbal interpretation of it:

I didn’t get given a number but he did say it wasn’t good news.

The form in which feedback is provided was perceived as important, as EBEs who had received more detailed feedback felt more confident leaving their appointment, and were more able to use this feedback to facilitate behavior change. In contrast, 1 EBE who received more limited feedback on the meaning of their score described feeling worried about their prognosis, describing feeling “in a lonely place” and “left in the lurch” following the appointment.

For the KLIFAD intervention, we understood these discussions as confirmatory of our model of providing standardized scripted feedback.

##### Sensitizing Concept 2: Receiving a Numeric Marker Can Provide a Target That Motivates and Rewards Recovery

EBE unanimously agreed that receiving a numeric marker of liver disease severity would enhance patient understanding, as long as this value was provided with context and scaling.

Some EBE described how numeric liver stiffness values received through a series of FibroScans motivated them to make behavioral changes to improve their liver health, and provided a reward as their liver became healthier:

So I started at 29 and the next time I went it was 24 and the next time I went it was 19 and then it was 17. So for me the numbers was a real motivation because I could see them going down.

This quote suggests a possible mechanism of action for interventions providing access to disease markers, for example, enabling change in health by providing an unambiguous target.

For the KLIFAD intervention, these discussions informed a decision to include the precise FibroScan value in the standardized scripted feedback and to augment it with material to aid interpretation.

##### Sensitizing Concept 3: Visual Representation of Disease Stage Can Aid Interpretation

EBE felt it was helpful to provide a visual representation of their liver stiffness result to help contextualize the numeric score. There was consensus for providing (1) a diagram of the liver with evidence of disease progression, to illustrate damage caused to the liver by excessive alcohol consumption; (2) a traffic light system (green being no significant damage detection, yellow being evidence of fibrosis, and red being evidence of cirrhosis) as a rapid means of presenting severity of liver disease progression. Other suggestions included (3) visual representations of the physical complications caused by advanced liver disease and (4) visual representations of alcohol use guidelines, for example, on low-risk drinking levels [24]. These specific suggestions were enacted in graphic design work conducted for the standardized scripted feedback.

##### Sensitizing Concept 4: Alcohol Misuse Can Be Associated With Impaired Capacity to Process Information

EBE felt that there was a balance to be struck between providing enough information that patients have a clear understanding of the risk of complications, their next steps, and guidance on alcohol use reduction, alongside being written at a level that is accessible for most readers. Further, 1 participant noted that this is particularly true for individuals experiencing alcohol misuse, who may feel overwhelmed by excessive information and may need important messages and medical terminology to be explained or simplified.

And obviously, in the midst of an addiction and dependency, you need it as simple as possible.

Some participants suggested that people attending treatment settings might be more likely to find reading difficult.

A proposed final version of standardized scripted feedback was reviewed by focus group attendees, to provide an opportunity for people with lived experience of alcohol-related difficulties to identify elements that might be problematic for others.

##### Sensitizing Concept 5: KAWs Routinely Tailor Information Presentation

KAWs validated an approach in which 3 scripts were produced and tailored to the disease state, and in which visual representations of the disease stage, alcohol use guidelines, and physical complications were included. However, they observed that an important part of their role was to use their judgment to decide how much information to impart to a patient, and to decide how to segment information across multiple sessions, suggesting the importance of retaining human judgment around information delivery in an otherwise standardized intervention. Operators described how a nonjudgemental, empathetic approach by the operator to their interactions with a patient was more likely to support recovery. In the KLIFAD trial of the KLIFAD intervention, operators were expected to use their judgment when delivering scripted feedback, for example, to adapt their presentation to the needs of participants, including by offering more detail on specific elements if needed.

### RA2: Production of Alcohol Misuse Recovery Narratives

#### Overview

At the PPI meeting which validated the video production script (Multimedia Appendix 3), members made recommendations summarized in Textbox 2.

**Recommendations on the integration of video recovery narratives into the KLIFAD (does knowledge of liver fibrosis affect high-risk drinking behavior) intervention.**

- The impact of alcohol misuse recovery narratives might be enhanced if accompanied by videos presenting accounts from caregiver and clinicians as well.
- FibroScan recipients may not feel fully informed about what a FibroScan is, even if they have received patient information.
- Receiving a high FibroScan score can be shocking, and in-person support might be needed by some after receiving a video.
- Some people may not be ready to receive videos directly after receiving a FibroScan and may need to access videos when they feel ready.
- Some people may need support from a psychotherapist to enable impact from the videos.
- Some people may be digitally excluded, and having the technology to access cannot be assumed.
- Some people can experience stigma about alcohol use through membership in a religious community, and this would be an important topic for a video.

There was a discussion, but no consensus, on whether videos should provide descriptions of local drinking cultures or be nationally relevant.

#### Form and Content of Videos

In total, 10 recovery narrative videos were produced. The videographer used their expertise to select from and modify the sample questions presented in Textbox 1. Each video began with an opening screen providing biographical details consisting of age, peak alcohol consumption, and initial FibroScan score. It concluded with a closing screen with an indication of the narrator’s current status. Video duration was from approximately 2 to 7 minutes. Settings were chosen in negotiation with the participant. Subtitles are present in English in all videos to enable accessibility for people with hearing impairment. Table 1 summarizes the key characteristics of narrators appearing in videos.

**Table 1:** Key characteristics of recovery narrative videos.

ID	Gender	Age (years)	Peak^a^	Score^b^	Disease state	Location^c^	Duration (min:s)
1^d^	Female	54	280	3.6	No fibrosis	Clinical	4:29
2	Female	46	210	12	Intermediate fibrosis	Home	4:28
3	Female	48	160	19	Cirrhosis	Clinical	2:53
4	Male	72	105	15	Cirrhosis	Clinical	4:07
5	Male	72	190	11	Intermediate fibrosis	Clinical	7:37
6	Male	53	190	>14	Cirrhosis	Clinical	3:59
7	Female	49	230	14	Intermediate fibrosis	Home	5:37
8	Male	52	140	15	Cirrhosis	Clinical	5:41
9	Male	68	200	4	No fibrosis	Clinical	4:37
10	Female	55	300	27	Cirrhosis	Clinical	5:17

^a^Peak is the approximate maximum number of units of alcohol consumed per week.

^b^Score is the first FibroScan score received by the narrator, as recalled by the narrator.

^c^Location is the setting for the video, as selected by the participant.

^d^IDs have been generated for reference in this paper. They do not match participant IDs collected during the research, and hence, data in this table should be thought of as anonymous rather than pseudonymous.

Participant 3 identified as being a member of a religious community. They chose to have their face obscured and to be pseudonymized due to fear of stigma from their community. All other participants chose to be identifiable in their video, including by being named and through not having their faces obscured. Participant 3 identified as Asian or Asian British. All other narrators identified as White.

Narrators of videos in the KLIFAD collection gave consent for their specific use in alcohol treatment settings. We did not ask for general consent for public use so as not to deter potential narrators from participation, given the persisting stigma around alcohol misuse [25]. However, 2 sample videos have been published with additional informed consent from narrators who are also PPI consultants to the KLIFAD study and who have previously shared their own accounts in public [26,27]. These demonstrate the production approach for the KLIFAD collection.

Informed by the PPI recommendations above, an additional video presenting a caregiver account was produced (the caregiver was 18 years or older, identified as a caregiver for someone meeting the criteria for an alcohol misuse recovery video participant, caring activities were not conducted as part of a health or social care profession). Further, informed by PPI recommendations, a video presenting clinical knowledge was produced (the clinician was 18 years or older and has cared for someone meeting the criteria for an alcohol misuse recovery video participant as part of a health care profession). Production of caregiver and clinician videos was less structured; the videographer worked with caregivers and a clinician to document material that they felt was relevant to an understanding of recovery from alcohol misuse.

The clinician video was 3 minutes long in a clinical setting. It described how liver disease can develop silently through the build-up of fat that causes scarring, and that liver failure can occur without warning. It described technologies to provide rapid diagnosis of problems, the choices that people can make after a diagnosis, and an optimistic future that can be experienced through changes in drinking behavior [28].

The caregiver video was 4 minutes long, set in the countryside. It featured a participant who was both a caregiver for a partner who had experienced alcohol misuse and someone who had recovered from alcohol misuse themselves. It described shared drinking behaviors, a sudden liver failure on the part of their partner, and a shared endeavor to stop alcohol use, including helping their partner manage difficulties relating to alcohol withdrawal such as hallucinations and physical shaking.

### Final Intervention Description

The final documents defining scripted feedback are in Multimedia Appendices 4 (no fibrosis), 5 (intermediate fibrosis), and 6 (cirrhosis). These were used in the feasibility RCT. During the trial, all videos were made available in the treatment setting, and accessed using tablet computers owned by the treatment provider. This includes video recovery narratives, a video presenting a caregiver perspective, and a video presenting a clinician perspective. Participants were invited to engage with videos after receiving TE, and as frequently as they wish on return to the same setting (eg, to access subsequent treatment sessions as part of allocated usual care). The reason for restricting access to the treatment setting is that prior mental health research suggests that accessing recovery narratives can cause distress [12], a finding which was amplified by PPI contributors when discussed in relation to KLIFAD videos. In the treatment setting participants could access in-person support from a KAW if needed. The consequences of restricting access to videos will be evaluated through the process evaluation of the KLIFAD trial, and this decision will be revisited.

## Discussion

### Principal Findings

We have presented knowledge developed through the intervention development process for the KLIFAD intervention. The end product was (1) a set of scripts to be used flexibly by FibroScan operators, (2) a video production guide and 12 associated videos (10 recovery narratives, 1 caregiver narrative, and 1 clinician narrative), and (3) decisions on how this material was to be integrated into a treatment setting. The caregiver and clinician narratives were unplanned and were the product of PPI recommendations. While narrators of recovery narratives were not specifically asked to discuss their experiences of receiving a FibroScan, in practice all did. Developers of a future iteration of the video production guide may wish to consider whether to make this a mandatory topic.

For the KLIFAD trial, videos were only made available in the treatment setting so that practical support could be provided in case of need, for example, if the video material elicited distress. These decisions will be evaluated through the KLIFAD trial process evaluation, which will be reported elsewhere. Focus group discussions provided preliminary evidence that a FibroScan score can create change by providing a numeric target that motivates and rewards recovery. This is a candidate mechanism that might be transferable to other interventions in which markers relevant to health are shared with patients at risk of related health conditions.

### Relationship to Previous Work

While for KLIFAD, we decided to limit video access to the treatment setting, other studies using recovery narratives have made different choices. The Narrative Experiences Online study has provided web-based access to a collection of mental health recovery narratives but has integrated a range of strategies to support the safety of users, for example, in relation to potentially distressing content. Unlike KLIFAD, narratives were mostly collected (with consent) from the public domain. Safety strategies include the use of content warnings for narratives with potentially distressing content; a user profile enabling users to block all narratives with specific forms of potentially distressing content; an “I’m upset” page containing self-management strategies and service signposting information in the event of distress [16]. The self-management and recovery technology study developed a web application providing access to videos describing psychosis experiences and psychosis recovery. Like KLIFAD, these were specifically produced for this study. Participants (N=10) in a feasibility trial accessed the web application in a series of colocated treatment sessions with mental health workers, and could optionally access the same web application outside of treatment sessions. All 10 participants indicated a positive emotional impact of the material, and none indicated a negative emotional impact [29]. Collectively, these 2 examples suggest that there is no ideal choice around the relationship between access to recovery narratives and user distress and that decision-making might be considered conditional on the context of use and narrative content. For the KLIFAD intervention, there is the potential for flexible models to be needed; some service users may wish to engage with recovery narratives immediately after receiving score and scripted feedback, while others may wish to return once ready.

### Strengths and Limitations

A strength of this study is that it incorporated 2 forms of expertise in its research process: EBE (of alcohol misuse and of the impact of receiving a FibroScan) and trained FibroScan operators. Our findings document their influence on the final form of the intervention integrated into our trial. Another strength is that this study has produced materials enabling the replication of the KLIFAD intervention. A limitation of this study, from the perspective of knowledge generation, is that processes were designed with the primary purpose of developing an intervention that was ready for deployment in a feasibility trial. This meant prioritizing the practical necessities of collecting feedback through focus groups and producing sufficient video narratives within a constrained timeframe; knowledge production might have been enhanced through larger sample sizes, requiring a longer research process.

### Implications for Practice and Research

Our work has drawn attention to some critical issues that might be attended to by developers of related interventions, such as those in which markers of disease or health are revealed to patients. These include (1) finding a balance between materials that are standardized on evidence-based good practices, while retaining sufficient freedom on the part of health workers to provide for a flexible, empathetic engagement with patients; (2) identifying strategies for supporting people who feel distressed if markers reveal unanticipated or substantial disease; and (3) producing narrative material that is authentic and impactful, while protecting narrators from the possible harms of identity disclosure. In our work, we have chosen a basic approach of tailoring materials purely to the disease state using a static model, but health research studies have frequently argued for the benefits of more dynamically tailoring materials and interventions to a broad range of individual characteristics and needs [30]. Developers of related interventions may consider how best to tailor materials that they provide to the populations of use.

### Conclusions

We have described the development process and associated knowledge products for the KLIFAD intervention, which consists of rapid access to a score providing a marker of liver disease, along with tailored operator feedback and access to a collection of recovery-oriented video narratives. Developers of related interventions, for example, those making use of biomarkers to create beneficial change in a recipient, should consider how best to present biomarkers so as to support and maximize the beneficial cognitive and behavioral changes that are created.

## Appendix

Multimedia Appendix 1 Focus group topic guide—experts by experience.

Multimedia Appendix 2 Focus group topic guide—key alcohol workers.

Multimedia Appendix 3 Final version of video production guide.

Multimedia Appendix 4 Scripted feedback—no fibrosis.

Multimedia Appendix 5 Scripted feedback—intermediate fibrosis.

Multimedia Appendix 6 Scripted feedback—cirrhosis.

## Abbreviations

ARLD: alcohol-related liver disease
EBE: experts by experience
KAW: key alcohol worker
KLIFAD: does knowledge of liver fibrosis affect high-risk drinking behavior?
PPI: patient and public involvement
RA: research activity
RCT: randomized controlled trial
TE: transient elastography
