# Supplementary material for: Transient Elastography and Video Recovery Narrative Access to Support Recovery From Alcohol Misuse: Development of a Novel Intervention for Use in Community Alcohol Treatment Services
Source: JMIR Form Res. 2023 Oct 4;7:e47109. doi: 10.2196/47109 (PMC10585443; doi:10.2196/47109)
Supplement: Multimedia Appendix 1 [file formative_v7i1e47109_app1.docx]

# WP1 Draft Topic Guide (For piloting) with Patients

***Study title:* Does knowledge of liver fibrosis affect high risk drinking behaviour (KLIFAD)? A feasibility randomised controlled trial**

**To begin**

Welcome to the focus group session. Thanks for taking the time to join us to talk about liver disease screening.

You were invited here today because you attended a liver scan appointment and were given your level of risk for liver disease using a Fibroscan machine. We would like to understand how to provide the best experience for patients undergoing the scan. This includes how the person operating the Fibroscan machine discusses the scan itself and then delivers the results of the scan to patients. We will ask you read through a script we have prepared to help operators talk through the scan and also a document that provides patients with their results.

Everyone’s risk of liver disease may be different. Because everyone has very different life experiences, there are no wrong answers to these questions, but rather differing points of view. Please feel free to share your point of view even if it differs from what others have said. Keep in mind that we're just as interested in negative comments as positive comments, and at times the negative comments are the most helpful.

*Logistics*

- Focus group will last about 2 hours

*Ground Rules*

- Hope that everyone feels comfortable enough to participate.
- Information provided in the focus group must be kept confidential
- This is an opportunity to help contribute to the treatment of liver disease!

CONSENT TO TAPE RECORD: I’m tape recording the session because I don't want to miss any of your comments. People often say very helpful things in these discussions and I can't write fast enough to get them all down.

If you talk about anyone else during the focus group by name (such as a friend or member of staff) – then we will keep their name anonymous when we write up the results by providing them with a false name. Likewise (the participant) we will also keep your identity anonymous during the write-up by giving you a false name in any reports resulting from this study

Are you okay with this? Do you have any questions?

- - Answer any questions they have
  - If they do not want to participate, thank them for their time and escort them out of venue. If they have participated via telephone or over video conferencing – finish the call.

**Beginning the focus group**

*Start recording the interview on the Dictaphone.*

Firstly, I want you to think back to your liver scan appointment.

1. Did you understand why you were undergoing a fibroscan and what the scan involved?
2. What was your experience of the scan? Was there anything about the way the operator conducted the scan or talked to you about the scan that you liked/disliked/found helpful?
3. After the scan, what information were you provided with? Including your results, any feedback from the scan operator, and any other information about liver disease?
   1. Was any of this difficult to understand? What information did you find most helpful?
4. Did the scan and/or scan results prompt you to make some changes to improve your liver health?
   1. If you received normal scan results, did you still want to make lifestyle changes?

Now I’d like us to spend the rest of the session today reviewing the documents in front of you. Please take some time to read through these documents and write any thoughts you have about the wording or how the information is presented on the document.

*Provide participants with pens*

*Give participants approximately 10-15 minutes to read through script and fibroscan results*

Let’s review the operator script. Imagine you were receiving this information from a fibroscan operator.

1. Do you understand the information presented in the script?
   1. What did you like/dislike about the script? What information was helpful/unhelpful? Was anything unclear?
2. Was there any information you felt was missing or that you think would make a useful addition to the script?
   1. Do you have any suggested changes or improvements to the script?
3. Do you think the results made sense for each level of liver disease stiffness?
   1. Did you understand the information? What information was helpful/unhelpful? Was anything unclear?
4. How did the documents make you feel?
   1. Did anyone have a negative reaction/positive reaction?
5. Did you like the way the results were presented (e.g. graphically, visually)?
   1. What would you change? Would you prefer the results to be presented as a value, on a scale, on a graph etc.?
6. Would you feel confident knowing what your result was and how to go about making lifestyle changes from this information?
   1. If not, why and what could we include that would help improve your confidence? Do you think the results documents would need explaining further by the operator?
7. Does anyone have additional thoughts about a specific result document (normal, likely fibrosis, likely cirrhosis)?
   1. Do you think the information reflects the level of risk and need for behaviour change?
8. Is there any other information we should include in the results document?
   1. Do you have any suggested changes or improvements to the results?

**Close**

Okay that reaches the end of the questions I wanted to ask today. Is there anything else you wanted to add or talk about that we didn’t talk about today?

If you’re okay to end the focus group there, I’ll switch the Dictaphone off, thank you!

**Debriefing**

- Thank you for speaking to us.
- Provide participants with sheet which outlines range of services etc, go through it with them. If there is any particular service/resource that they have expressed an interest in – then signpost them to it.
  - If they have participated via telephone– state that they can be sent this via email if this wish or it can be read out to them.
- Thank them again, and ask if they are feeling okay to leave the building/ or hang up/exit the call.
