# Supplementary material for: Transient Elastography and Video Recovery Narrative Access to Support Recovery From Alcohol Misuse: Development of a Novel Intervention for Use in Community Alcohol Treatment Services
Source: JMIR Form Res. 2023 Oct 4;7:e47109. doi: 10.2196/47109 (PMC10585443; doi:10.2196/47109)
Supplement: Multimedia Appendix 2 [file formative_v7i1e47109_app2.docx]

# WP1 Draft Topic Guide (For piloting) with Fibroscan Operators

***Study title:* Does knowledge of liver fibrosis affect high risk drinking behaviour (KLIFAD)? A feasibility randomised controlled trial**

**To begin**

Welcome to the focus group session. Thanks for taking the time to join us to talk about liver disease screening.

You were invited here today because you currently provide or have previously provided fibroscans to identify liver disease. We would like to understand how to provide the best experience for patients undergoing the fibroscan. This includes standardising how the health care professional operating the fibroscan machine discusses the scan itself and then delivers the results of the scan to patients. We will ask you read through a script we have prepared to help operators talk through the scan and also a document that provides patients with their results. We are particularly interested in how you would feel delivering the operator script and results, and whether you see any barriers to implementation.

There are no wrong answers to these questions, but rather differing points of view. Please feel free to share your point of view even if it differs from what others have said. Keep in mind that we're just as interested in negative comments as positive comments, and at times the negative comments are the most helpful.

*Logistics*

- Focus group will last about 1.5 hours
- Feel free to move around
- Where is the bathroom? Exit?
- Help yourself to refreshments

*Ground Rules*

- Hope that everyone feels comfortable enough to participate.
- Information provided in the focus group must be kept confidential
- Stay with the group and please don’t have side conversations
- Turn off mobile phones if possible
- This is an opportunity to help contribute to the treatment of liver disease!

You've probably noticed the microphone. I’m tape recording the session because I don't want to miss any of your comments.

If you talk about anyone else during the focus group by name (such as a colleague) – then we will keep their name anonymous when we write up the results by providing them with a false name. Likewise (the participant) we will also keep your identity anonymous during the write-up by giving you a false name in any reports resulting from this study

Are you okay with this? Do you have any questions?

- - Answer any questions they have
  - If they do not want to participate, thank them for their time and escort them out of venue. If they have participated via telephone or over video conferencing – finish the call.

**Beginning the focus group**

*Start recording the interview on the Dictaphone.*

We have provided you with the operator script and three sets of results for normal, likely fibrosis, and likely cirrhosis fibroscan results. Please take some time to read through these documents and write any thoughts you have about the wording or how the information is presented on the document.

*Provide participants with pens*

*Give participants approximately 10-15 minutes to read through script and fibroscan results*

Let’s review the operator script. Imagine you were giving this information to patients attending their fibroscan appointment.

1. Was the script clear?
   1. Would it be easy to deliver? Would you feel comfortable delivering this script?
2. What did you like/dislike about the script?
   1. What information was helpful/unhelpful? Was anything unclear to you as the operator?
3. Was there any information you felt was missing or that you think would make a useful addition to the script?
4. Can you see any potential barriers to delivering the script in your workplace (e.g. time constraints, lack of comfort with material)?
   1. Do you have any suggestions for how to overcome these barriers? Would you need any additional training or guidance?

Now let’s review the fibroscan result documents. There are three different results a patient can receive, depending on their liver stiffness. Imagine you were giving this information to patients attending their fibroscan appointment.

1. Do you think the results made sense for each level of liver disease stiffness?
   1. Would they be easy to deliver? Would you feel comfortable providing patients with these results?
   2. What information was helpful/unhelpful? Was anything unclear?
2. Is there anything you would change about the way the results are presented?
   1. Do you think patients would understand the results? Do you think the results documents would need further explanation?
3. Does anyone have additional thoughts about a specific result document (normal, likely fibrosis, likely cirrhosis)?
   1. Is there any other information we should include in the results document?
4. Can you see any potential barriers to delivering the results in your workplace (e.g. time constraints, lack of comfort with material)?
   1. Do you have any suggestions for how to overcome these barriers? Would you need any additional training or guidance?

**Close**

Okay that reaches the end of the questions I wanted to ask today. Is there anything else you wanted to add or talk about that we didn’t talk about today?

If you’re okay to end the focus group there, I’ll switch the Dictaphone off, thank you!

**Debriefing**

- Thank you for speaking to us, your feedback will help us to optimise the way patients receive feedback following their fibroscan.
