# Supplementary material for: Transient Elastography and Video Recovery Narrative Access to Support Recovery From Alcohol Misuse: Development of a Novel Intervention for Use in Community Alcohol Treatment Services
Source: JMIR Form Res. 2023 Oct 4;7:e47109. doi: 10.2196/47109 (PMC10585443; doi:10.2196/47109)
Supplement: Multimedia Appendix 3 [file formative_v7i1e47109_app3.docx]

KLIFAD Recovery Video Story Production Guide

# Introduction

Alcohol-related liver disease is a major cause of early death; it often has no symptoms in the early stages. Consequently, people are frequently diagnosed once there is already a build-up of scar tissue in the liver, which can lead to liver failure. Fibrosis is the medical term for scarring of the liver. FibroScan is a type of ultrasound that measures the degree of stiffness in the liver caused by this scarring.

The KLIFAD study, starting in June 2021 will make FibroScan available for free to more than 100 **trial participants** in Nottinghamshire. This will help us learn about the feasibility of adding FibroScan results to existing advice given to people with excess alcohol use. After receiving a FibroScan, trial participants will receive a standard medical information sheet explaining their results. They will also receive access to videos presenting stories of recovery from excess alcohol use. These videos will be available in the clinic (such as on an iPad) and also subsequently at home, such as through a web-page.

We are creating videos during KLIFAD by working with people who have a history of excess alcohol intake and who have received a FibroScan (this is our primary focus), and also with people who identify as carers for someone with a history of excess alcohol intake. Both are referred to as **video participants**. Our aim will be to produce videos that have the maximum beneficial impact on the alcohol use of trial participants (e.g. the idea is that the video contributes to the person substantially reducing or ceasing their alcohol use).

# Recovery Definitions

In context of Alcohol Use Disorder (AUD) recovery has been defined in variety of ways; Katie Wiltkiewitz et al recognise recovery in AUD as a period of sustained abstinence from alcohol and improvement in social and employment functioning, and mental and physical health (1).

The UK Drug Policy commission define recovery in substance misuse as ‘‘voluntarily sustained control over substance use, which maximises health and wellbeing and participation in the rights, roles and responsibilities of society’(2).

The Betty Ford institute consensus panel define recovery in Alcohol and substance misuse as ‘a voluntarily maintained lifestyle composed characterized by sobriety, personal health, and citizenship’ (3). The components of this definition are elaborated as; **Sobriety:** refers to abstinence from alcohol and all other nonprescribed drugs. **Personal health:** refers to improved quality of personal life as defined and measured by validated instruments such as the physical health, psychological health, independence, and spirituality scales of the World Health Organization QOL instrument. **Citizenship:** Citizenship refers to living with regard and respect for those around you as defined and measured by validated instruments such as the social function and environment scales of the WHO-QOL instrument

The U.S. Department of Health & human services Substance abuse and mental health service administration (SAMHSA) define recovery in substance use disorder as ‘A process of change through which individuals improve their health and wellness, live a self-directed life, and strive to reach their full potential’(4).

This definition has much in common with definitions of recovery in mental health research, which describe recovery as “A deeply personal, unique process of change, a way of living a satisfying, hopeful and contributing life even with limitations caused by illness [and] a process involving the development of new meaning or purpose in one’s life” (5, 6).

# Inclusion and exclusion criteria: AUD video participants

## Inclusion Criteria

- A person age 18 years and over attending with a primary problem of alcohol misuse as defined by initial clinical assessment
- Is certain in their belief to have experienced recovery from their alcohol-use condition and/or has been identified as having experienced recovery by the study team against any of the alcohol-related criteria above
- Has capacity to give informed consent i.e. is fit and able to voluntarily agree to take part in this study
- Willing to be recorded for video
- Has previously received one or more FibroScans
- Can recall and are willing to share with the research team their first FibroScan score, or can identify an approximate value for their score

# Inclusion and exclusion criteria: Carer video participants

## Inclusion criteria

- Adults (18+) of any gender
- Identifies as a carer for someone meeting the criteria for an AUD video participant

## Exclusion criteria

- Caring activities have been as part of their profession (e.g. as a nurse or doctor)

# Sampling method

For AUD video participants, we will aim for a maximum variation sample on the category of the first FibroScan score received by the video participant (Tashakkori & Teddlie, 2003a). Scores reported by participants will be allocated to three categories (representing low, moderate and high scores) and we will aim to include two participants per category.

Where possible, we will include participants with a range of genders and ethnicities (both as self-reported), but only if this does not disrupt the maximum variation sample above.

# Ethical considerations

Informed consent will be collected by Mohsan Subhani or delegate before video recording begins, in keeping with the study protocol. Final videos should not include full names of the participant or names of third parties. The latter holds even for carers, in case of future relationship breakdown.

If, during the interview, a video participant reveals the name of a third party, then that part of the interview should be re-shot with instructions not to use the third party name, and the segment with the name of the third party should not be used in the final video.

A proposed final video will be shown to the participant, to allow them to raise any objections to content. The proposed final video will be reviewed by the KLIFAD team to identify any content that might be thought to cause harm to people watching the video that needs removing (it is preferable NOT to remove any material).

# Principles of care for video participants

Adapted from a Scottish Recovery Network guide to narrative collection (7)

**Control**: Video participants should always be in control of the process. Their story should not be altered or adapted. They should be able decide what is shared, how it is shared and when it is shared.

**Support**: Video participants should be given the time and resources they need to think through their story and to decide what they want to share.~~.~~

**Respect:** Everyone’s lived experience and recovery journey is different. The experiences of video participants should be respected.

**Wellbeing:** Sharing stories is an empowering experience but it can feel emotional and challenging at times. The wellbeing of the video participant should always be at the centre of our considerations and be given as much time as they need to feel comfortable to proceed. A video participant has the write to withdraw from the filming if they feel that they are unable to start or continue

**Responsibility:** The video participant sharing their story is ultimately responsible for deciding what they want to share, when and with whom.

# Role of facilitator

The role of the facilitator is to:

- invite, respect and validate the experiences shared and highlighting strengths and skills identified during the process
- react to difficult feelings and behaviours with compassion and curiosity rather than irritation or defensiveness
- avoid making judgements
- be aware of narrator’s (video participant) own needs and how they can maintain their wellbeing
- create a positive and safe environment

Above all other considerations, videos produced during KLIFAD should present **authentic** accounts. This is because stories that are perceived as authentic by trial participants are more likely to make an impact on them. The facilitator should support video participants in providing authentic accounts.

# Interviewer direction

## AUD video participants

The facilitator should support the video participant in describing what their recovery from excess alcohol use has meant to them.

The facilitator should check with the video participant about whether receiving a FibroScan has had an impact on the video participant’s recovery (either positive or negative). If it has had an impact, then the facilitator should support the video participant in talking about the impact of the FibroScan in the video.

Some people are happy to talk about recovery, but others find the concept, or the word challenging and respond better to questions using terms such as wellbeing and having a good life. Some are happy to ponder the big questions, but others need more specific questions to help them get started.

As such, videos produced from this process might be a mixture of videos which A) start with single open-ended question, with the narrators (video participant) telling the story without interruption and B) consist of responses to questions asked during the interview.

NEON theory suggests that the following elements might make for a particularly impactful video.

- The narrator talks about their achievements in relation to alcohol use
- The narrator talks about their difficulties in relation to alcohol use
- The narrator talks about the impact of their alcohol use on others
- The narrator talks about the strategies that they have successfully used to recover
- The narrator talks about how thinking about alcohol use has changed
- The narrator talks about the barriers to their recovery they have experienced
- The narrator talks about the beliefs and values that have supported their recovery
- The narrator talks about their emotions around their alcohol use
- The narrator shares advice on how best to make use of health services to support recovery
- The narrator talks about the impacts they belief their alcohol use and behaviours had upon others and the attitudes arising from that e.g. their carer, other family members, friends etc.

These elements might guide the questions asked by the video facilitator. Some sample questions are below.

What does recovery mean to you?

- What gives you hope?
- What makes you feel well?

What has helped your recovery?

- What was your first step on your recovery journey?
- What helped you take this step?
- What works for you and why?
- What activities have helped you? How do you feel when you are doing them? What has helped during times of hardship?
- At what point did you realise that you needed support?
- Where did you find the support? Was this challenging?
- Has there been someone who has supported you during your recovery journey? What were the barriers to recovery? How did you overcome them?
- What has been unhelpful or missing in your recovery?

What have you learned about recovery?

- Do you have any techniques that have been helpful when you are feeling really down?
- What sort of lessons would you like to pass onto others?
- If you could give one thing to assist someone’s recovery what would that be?
- What has helped you to build resilience?
- What would you tell someone who feels they won’t recover?
- How did you deal with changes to your recovery journey?

## Carer video participants

Carers might be asked to talk through what it has felt like to be a carer, and how they have supported the person that they care for.

# Video specification

- 2-5 minutes
- Interview asks questions but does not physical appear in the video

# References

1. Witkiewitz K, Wilson AD, Pearson MR, Montes KS, Kirouac M, Roos CR, et al. Profiles of recovery from alcohol use disorder at three years following treatment: can the definition of recovery be extended to include high functioning heavy drinkers? Addiction (Abingdon, England). 2019;114(1):69-80.

2. <Policy report - A vision of recovery_ UKDPC recovery consensus group.pdf>.

3. What is recovery? A working definition from the Betty Ford Institute. Journal of substance abuse treatment. 2007;33(3):221-8.

4. <SANHSA'S working definition of Recovery.pdf>.

5. Anthony WA. Recovery from mental illness: The guiding vision of the mental health service system in the 1990s. Psychosocial Rehabilitation Journal. 1993;16(4):11-23.

6. Rennick-Egglestone S, Morgan K, Llewellyn-Beardsley J, Ramsay A, McGranahan R, Gillard S, et al. Mental Health Recovery Narratives and Their Impact on Recipients: Systematic Review and Narrative Synthesis. Canadian journal of psychiatry Revue canadienne de psychiatrie. 2019;64(10):669-79.

7. <MRR_Facilitator_Guide.pdf>.

8. Prochaska JO, Velicer WF. The transtheoretical model of health behavior change. American journal of health promotion : AJHP. 1997;12(1):38-48.

9. Slade M, Amering M, Farkas M, Hamilton B, O'Hagan M, Panther G, et al. Uses and abuses of recovery: implementing recovery-oriented practices in mental health systems. World psychiatry : official journal of the World Psychiatric Association (WPA). 2014;13(1):12-20.

10. Best DW, Lubman DI. The recovery paradigm - a model of hope and change for alcohol and drug addiction. Australian family physician. 2012;41(8):593-7.

11. Rennick-Egglestone S, Ramsay A, McGranahan R, Llewellyn-Beardsley J, Hui A, Pollock K, et al. The impact of mental health recovery narratives on recipients experiencing mental health problems: Qualitative analysis and change model. PloS one. 2019;14(12):e0226201-e.

12. STOTLAND NL, MATTSON MG, BERGESON S. The Recovery Concept: Clinician and Consumer Perspectives. Journal of Psychiatric Practice®. 2008;14:45-54.
