# Supplementary material for: Transient Elastography and Video Recovery Narrative Access to Support Recovery From Alcohol Misuse: Development of a Novel Intervention for Use in Community Alcohol Treatment Services
Source: JMIR Form Res. 2023 Oct 4;7:e47109. doi: 10.2196/47109 (PMC10585443; doi:10.2196/47109)
Supplement: Multimedia Appendix 4 [file formative_v7i1e47109_app4.docx]

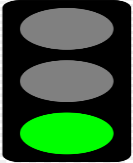


Title of the Study: Does knowledge of liver fibrosis affect high-risk drinking behaviour (KLIFAD)?

**Normal Fibroscan information to patients**

A feasibility randomised controlled trial.

Thank you for volunteering to have a Fibroscan today.

**Interpretation of results**

Fibrosis is the medical term for the scarring of the liver. FibroScan is a device that measures this scarring by estimating the stiffness of the liver. This stiffness is measured in units called Kilopascal or kPa. The score below gives an estimate of your liver fibrosis.

**kPa**

Your scan result was:


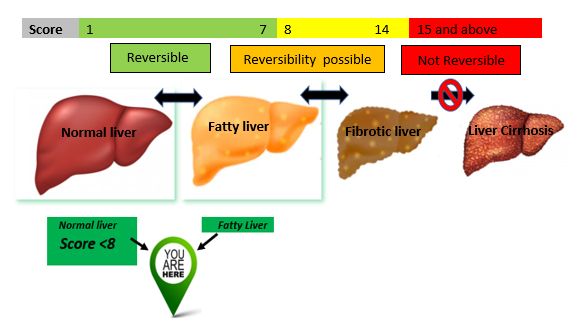


Your liver **stiffness score is low**. We wouldn't normally expect to see liver scarring with this score although you may **still have fatty liver and are at risk of developing liver disease** if you continue to drink.

**What risk is there to my health?**

Even though your FibroScan score is low, this does not mean you don’t have a liver problem, or won’t have liver problems in the future. 1 person in 10 who drink more than the Government recommended 14 units per week have fatty liver. Once you develop fatty liver, 1 person in 2.5 can develop advanced liver scarring and cirrhosis.

It is very important that you reduce or stop drinking alcohol completely. **If you can reduce or stop drinking alcohol, your risk of liver problems will be significantly reduced.** If you continue to drink heavily then you may be at risk of serious complications, such as **liver failure,** shown in the next image:

**
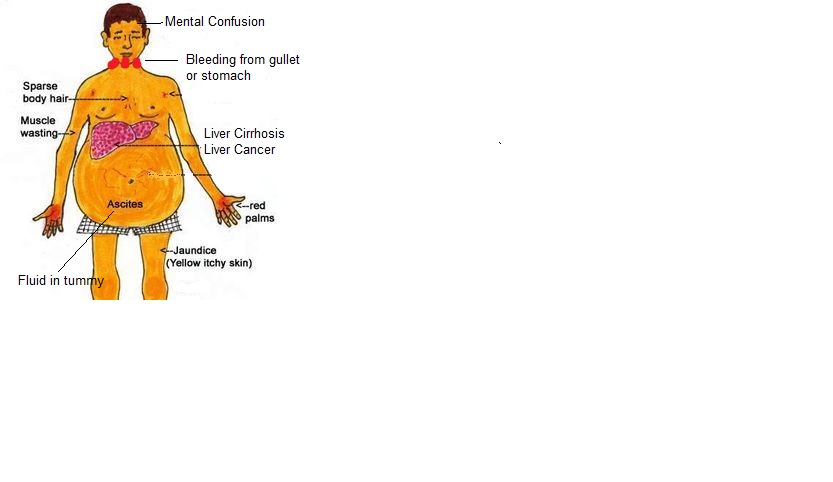
**

**Liver disease complications**

**Other potential health problems**


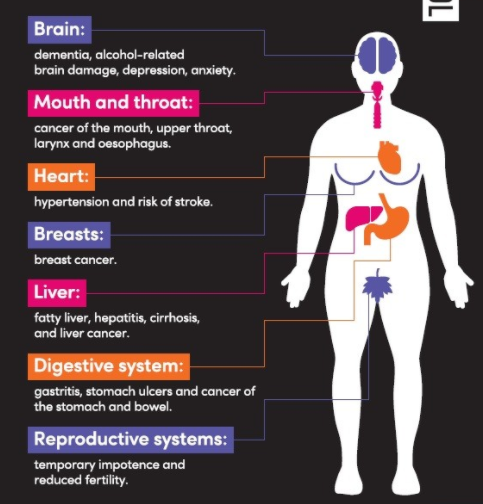
Drinking excessively can also impact your health in lots of other ways. The picture below shows other **long term risks of drinking excess alcohol:**

Source: https://alcoholchange.org.uk/

**Advice**

The good news is if you cut down your alcohol intake or become abstinent it will stop future damage to the liver and there is a chance that any existing damage to your liver can improve. The Government recommends that both men and women drink under 14 units of alcohol per week. This is equivalent to drinking no more than 6 pints of average strength beer (4%) or 7 medium-sized glasses of wine (175ml, 12%) per week. For some people**, it may be dangerous to stop drinking suddenly**, so we advise gradually reducing the amount you drink and discussing this with your key alcohol worker or GP.


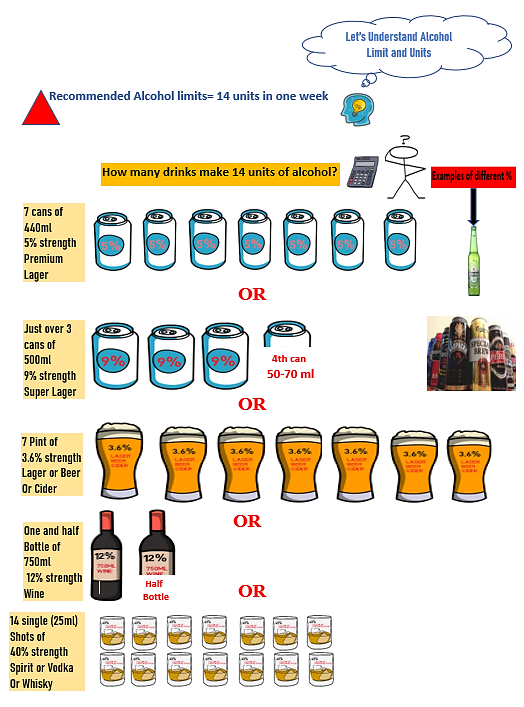


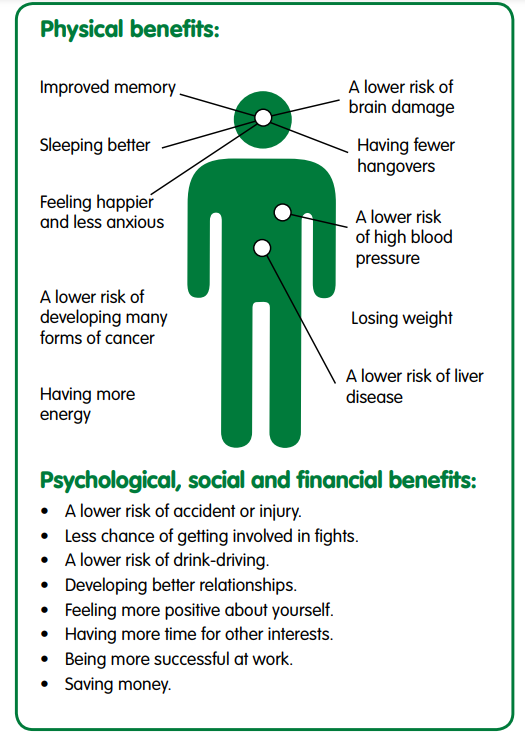


Source:<https://www.healthscotland.scot/media/3096/making-a-change-english-april2020.pdf>

**Links for more information on alcohol misuse and ways to find support**

If you are interested to read more on how alcohol can effect your health and what help is available please follow these links for more information

NHS alcohol misuse guide
<https://www.nhs.uk/conditions/alcohol-misuse/risks/>

Patient.info Alcohol dependence and Problem Drinking
<https://patient.info/healthy-living/alcohol-and-liver-disease/alcoholism-and-problem-drinking>

NHS Alcohol support
<https://www.nhs.uk/live-well/alcohol-support/>

Alcohol Change UK- Get help now
<https://alcoholchange.org.uk/help-and-support/get-help-now>

**Benefits of reducing or stopping alcohol**
