# Supplementary material for: Transient Elastography and Video Recovery Narrative Access to Support Recovery From Alcohol Misuse: Development of a Novel Intervention for Use in Community Alcohol Treatment Services
Source: JMIR Form Res. 2023 Oct 4;7:e47109. doi: 10.2196/47109 (PMC10585443; doi:10.2196/47109)
Supplement: Multimedia Appendix 5 [file formative_v7i1e47109_app5.docx]

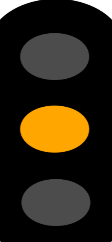
 Title of the Study: Does knowledge of liver fibrosis affect high-risk drinking behaviour (KLIFAD)? A feasibility randomised controlled trial.

**Intermediate (fibrosis) Fibroscan information to patients**

**Fibroscan Intermediate reading information to patients**

Thank you for volunteering to have a Fibroscan today.

**Interpretation of results**

Fibrosis is the medical term for the scarring of the liver. Fibroscan is a device that measures this scarring by estimating the stiffness of the liver. The stiffness is measured in units called Kilopascal or kPa.. The score below gives an estimate of your liver fibrosis.

kPa

You scan result was:


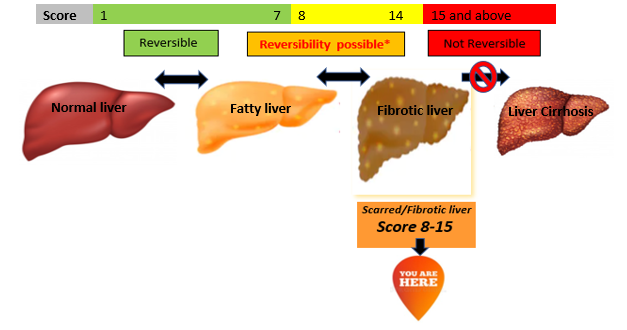


Your liver **stiffness score is high**. We would normally expect to see **moderate liver scarring** with this score.

**What risk is there to my health?**

You already have evidence of liver damage. If you continue to drink at the current level, there is a high risk (1 in 2.5 persons) you will progress to liver cirrhosis.

It is very important that you should reduce or stop drinking completely. **If you can reduce or stop drinking alcohol, your risk of liver problems is significantly reduced**. If you continue to drink heavily then you are at risk of serious complications, such as liver failure as shown in next image.

**Liver disease complications**


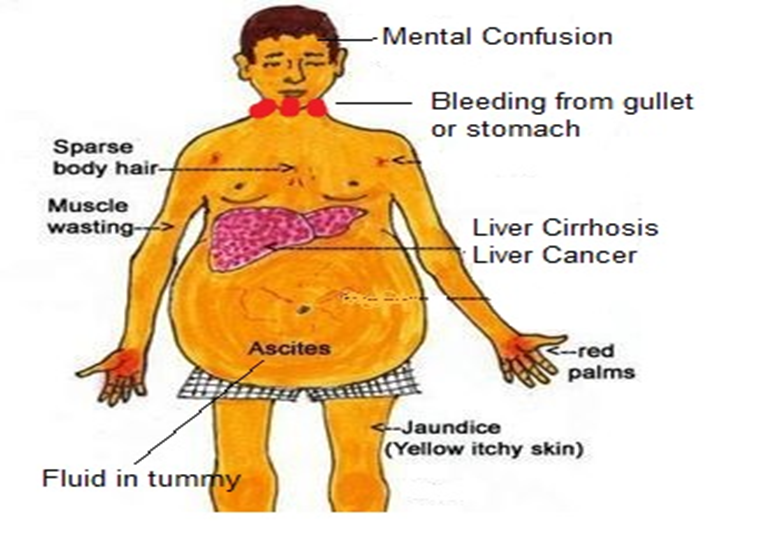


**Other potential health problems**


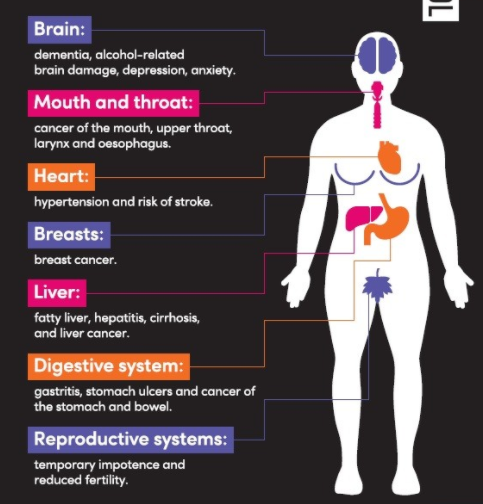
Drinking excessively can also impact your health in lots of other ways. The picture below shows other **long term risks of drinking excess alcohol:**

Source: https://alcoholchange.org.uk/

**Advice**

The good news is if cut your alcohol intake down or become abstinent it will stop future damage to the liver and there is a chance that any existing damage to your liver may improve. The Government recommends that both men and women drink under 14 units of alcohol per week. This is equivalent to drinking no more than 6 pints of average strength beer (4%) or 7 medium-sized glasses of wine (175ml, 12%). For some people**, it may be dangerous to stop drinking suddenly,** so we advise gradually reducing the amount you drink and discussing this with your key alcohol worker or GP.


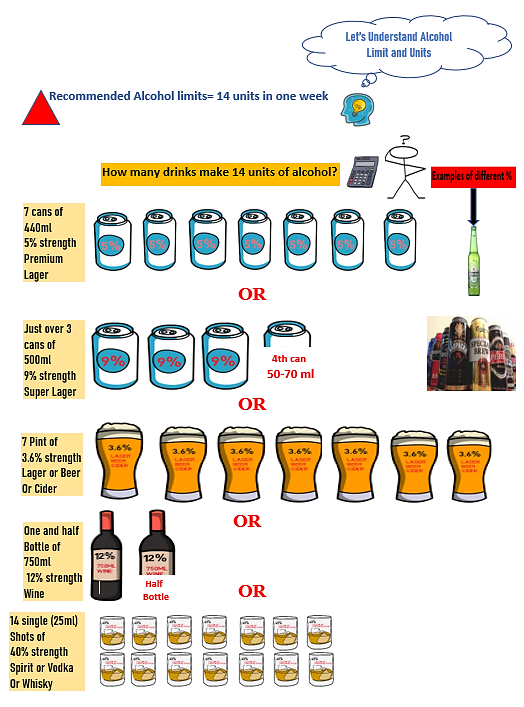


**Benefits of reducing or stopping alcohol**


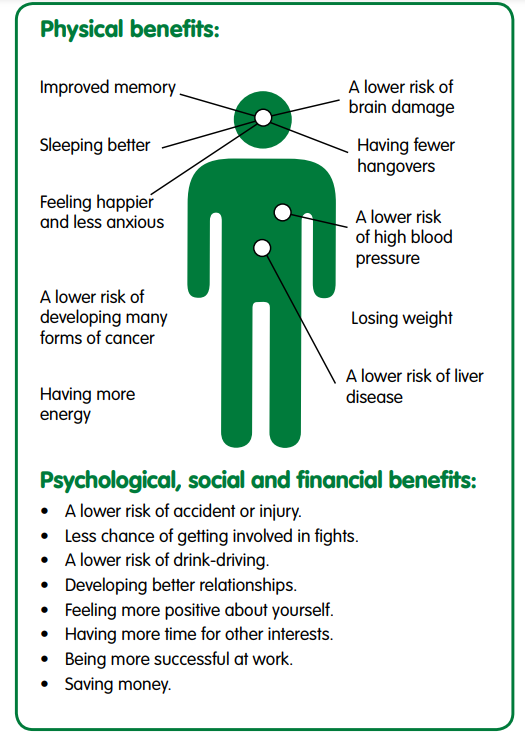


Source:<https://www.healthscotland.scot/media/3096/making-a-change-english-april2020.pdf>

**Links for more information on alcohol misuse and ways to find support**

If you are interested to read more on how alcohol can effect your health and what help is available please follow these links for more information

NHS alcohol misuse guide
<https://www.nhs.uk/conditions/alcohol-misuse/risks/>

Patient.info Alcohol dependence and Problem Drinking
<https://patient.info/healthy-living/alcohol-and-liver-disease/alcoholism-and-problem-drinking>

NHS Alcohol support
<https://www.nhs.uk/live-well/alcohol-support/>

Alcohol Change UK- Get help now
<https://alcoholchange.org.uk/help-and-support/get-help-now>
