# Supplementary material for: Transient Elastography and Video Recovery Narrative Access to Support Recovery From Alcohol Misuse: Development of a Novel Intervention for Use in Community Alcohol Treatment Services
Source: JMIR Form Res. 2023 Oct 4;7:e47109. doi: 10.2196/47109 (PMC10585443; doi:10.2196/47109)
Supplement: Multimedia Appendix 6 [file formative_v7i1e47109_app6.docx]

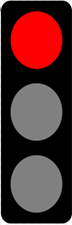


**Advanced (fibrosis) Fibroscan information for patients**

Title of the Study: Does knowledge of liver fibrosis affect high-risk drinking behaviour (KLIFAD)? A feasibility randomised controlled trial.

Thank you for volunteering to have a Fibroscan today.

**Interpretation of results**

Fibrosis is the medical term for the scarring of the liver. Fibroscan is a device that measures this scarring by estimating the stiffness of the liver. The stiffness is measured in units called Kilopascal or kPa. The score below gives an estimate of your liver fibrosis.


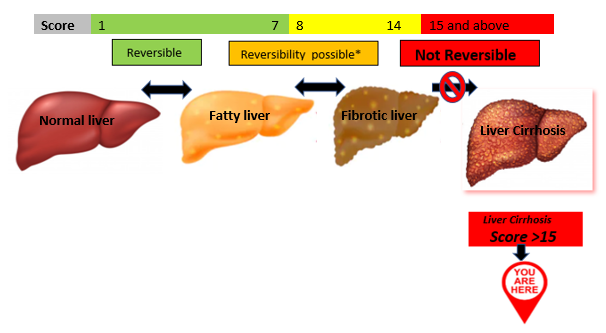
Your scan result was:

Your liver **stiffness score is high.** We expect to see **advanced liver scarring/Cirrhosis** with degree of liver stiffness.

kPa

**What risk is there to my health?**

Alcohol is one of the main causes of liver cirrhosis. If you continue to drink at the current level and do not abstain from alcohol, you have more than a **30% (1 person in 3)** chance of dying within **5 years.** Once you develop complications, the **risk of death within 5 years is as high as 65% (1 person in 1.5)**.

It is very important that you should stop drinking alcohol completely. **If you can stop drinking alcohol, your risk of future liver problems is significantly reduced.** If you continue to drink heavily then you are at risk of serious complications, such as liver failure as shown in next image. Liver failure has a huge impact on people, affecting their ability to live independently and increasing their risk of early death.


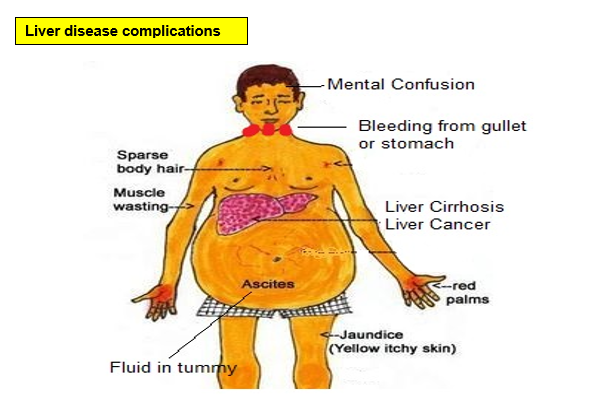


**Other potential health problems**

Drinking excessively can also impact your health in lots of other ways. The picture below shows other **long-term risks of drinking excess alcohol:**


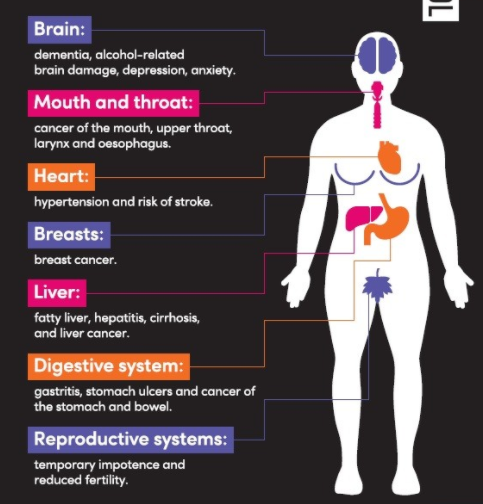


Source: https://alcoholchange.org.uk/

**Advice**


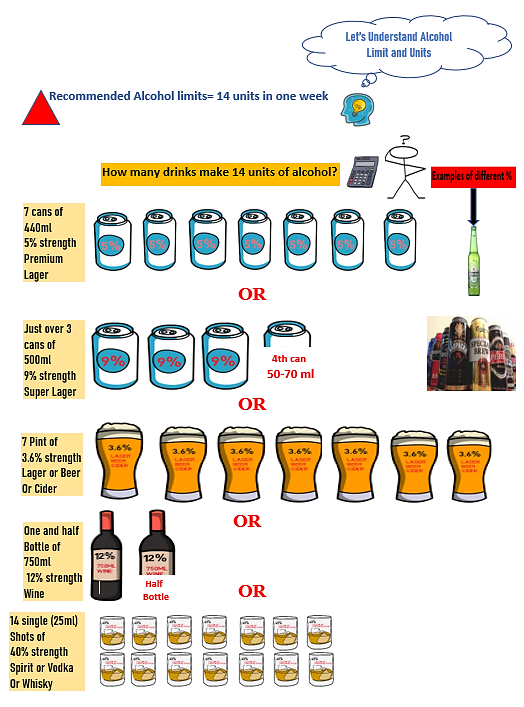
When liver damage is at this stage it is no longer reversible, however it is possible to prevent further damage and future complications. Our advice is to stop drinking alcohol permanently as this will help your liver to function well. For some people, **it may be dangerous to stop drinking suddenly**, so we advise gradually reducing the amount you drink and discussing this with your key alcohol worker or GP. We will also recommend that your GP refers you to the Nottingham University Hospital Liver team for further advice.

**Benefits of reducing or stopping alcohol**


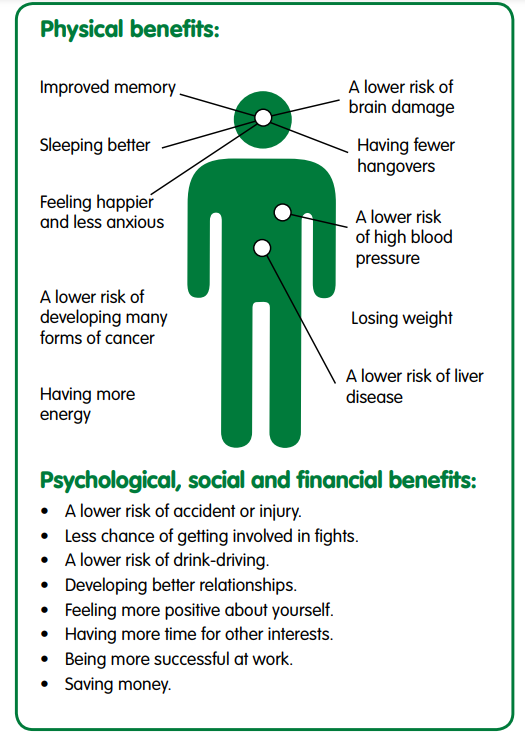


**Links for more information on alcohol misuse and ways to find support**

If you are interested to read more on how alcohol can affect your health and what help is available please follow these links for more information

NHS alcohol misuse guide
<https://www.nhs.uk/conditions/alcohol-misuse/risks/>

Patient.info Alcohol Dependence and Problem Drinking
<https://patient.info/healthy-living/alcohol-and-liver-disease/alcoholism-and-problem-drinking>

NHS Alcohol support
<https://www.nhs.uk/live-well/alcohol-support/>

Alcohol Change UK- Get help now
<https://alcoholchange.org.uk/help-and-support/get-help-now>

Source:<https://www.healthscotland.scot/media/3096/making-a-change-english-april2020.pdf>
